# Does interhospital transfer improve outcome of acute myocardial infarction? A propensity score analysis from the Cardiovascular Cooperative Project

**DOI:** 10.1186/1471-2261-8-22

**Published:** 2008-09-09

**Authors:** John M Westfall, Catarina I Kiefe, Norman W Weissman, Anthony Goudie, Robert M Centor, O Dale Williams, Jeroan J Allison

**Affiliations:** 1 University of Colorado Denver – Anschutz Medical Campus, Denver, Colorado, USA; 2 University of Alabama at Birmingham, Birmingham, Alabama, USA; 3 Associate Professor of Family Medicine, Dept of Family Medicine, A01, P.O Box 6511 – Mail Stop F496, Aurora, CO 80045

## Abstract

**Background:**

Many patients suffering acute myocardial infarction (AMI) are transferred from one hospital to another during their hospitalization. There is little information about the outcomes related to interhospital transfer. The purpose of this study was to compare processes and outcomes of AMI care among patients undergoing interhospital transfer with special attention to the impact on mortality in rural hospitals.

**Methods:**

National sample of Medicare patients in the Cooperative Cardiovascular Study (n = 184,295). Retrospective structured medical record review of AMI hospitalizations. Descriptive study using a retrospective propensity score analysis of clinical and administrative data for 184,295 Medicare patients admitted with clinically confirmed AMI to 4,765 hospitals between February 1994 and July 1995. Main outcome measure included: 30-day mortality, administration of aspirin, beta-blockers, ACE-inhibitors, and thrombolytic therapy.

**Results:**

Overall, 51,530 (28%) patients underwent interhospital transfer. Transferred patients were significantly younger, less critically ill, and had lower comorbidity than non-transferred patients. After propensity-matching, patients who underwent interhospital transfer had better quality of care anlower mortality than non-transferred patients. Patients cared for in a rural hospital had similar mortality as patients cared for in an urban hospital.

**Conclusion:**

Transferred patients were vastly different than non-transferred patients. However, even after a rigorous propensity-score analysis, transferred patients had lower mortality than non-transferred patients. Mortality was similar in rural and urban hospitals. Identifying patients who derive the greatest benefit from transfer may help physicians faced with the complex decision of whether to transfer a patient suffering an acute MI.

## Background

Ischemic heart disease is the leading cause of death worldwide, causing 6.26 million deaths per year[1]. Acute myocardial infarction (AMI) is a major cause of death in the United States, accounting for 203,551 deaths in 1998[2]. A growing number of AMI patients are transferred from one hospital to another during their hospital course[3,4].

While there is nothing intrinsically beneficial about moving a patient from one hospital to another, transfer may provide the opportunity for higher level of care and more advanced treatment. Several observational studies on general medical and surgical patients reported that transferred patients, regardless of their diagnosis, were sicker, had more co-morbid conditions, used more resources, required longer hospital stays, and had higher mortality [5-7]. Another study found that transferred patients had less severe illness and lower mortality[8]. Factors that might confound these previous findings include changing economic motivation for transfer, greater variation in availability of advanced technology, and widespread attempts to improve quality of care [3,9].

Early studies on myocardial infarction called for the transfer of "high risk patients"[10]. The conventional wisdom was to transfer the sickest cardiac patients or patients who had failed less invasive therapy to the tertiary care hospital for specialized care [11-14]. However, several more recent studies found that transferred acute MI patients were younger and had fewer comorbid conditions[4,15]. Rural MI patients are more likely to be transferred, however, rural patients have also been reported to receive lower quality of care[16]. Many studies on acute MI have deleted some or all transferred patients from their analysis [17-19].

The impact of interhospital transfer on processes and outcomes of acute MI has largely gone unstudied. Because the number of AMI patients undergoing interhospital transfer is rising we examined the impact of interhospital transfer on mortality. We used data from the Cooperative Cardiovascular Project (CCP), a large and representative sample with detailed clinical and quality of care information on patients hospitalized with AMI.

## Methods

### Cardiovascular Cooperative Project

The CCP was a national quality improvement project sponsored by the Centers for Medicare and Medicaid Services (CMS), formerly the Health Care Financing Administration for Medicare patients hospitalized with AMI[20,21]. Patients were initially identified from Medicare claims data using the principal diagnosis code of 410 from International Classification of Diseases, Ninth Revision, Clinical Modification[22]. The CCP performed structured medical record review for 234,769 Medicare fee for service patients randomly sampled from 6,684 hospitals in all 50 states who were hospitalized for AMI between February 1994 and July 1995. As a quality check, an independent abstraction for a randomly selected 5% of the charts was done to assess reliability and validity for key variables. The methods of the CCP are fully described elsewhere[20,21].

### Patients

Patients were excluded from our analyses for: 1) lack of clinically confirmed AMI according to criteria established by Ellerbeck[21] (n = 31,194); 2) admission to hospital with unclear teaching status, technology index, or rural/urban status (n = 262); 3) age less than 65 years (n = 15,072); and 5) death on day of admission for patients who were not transferred (n = 3,946). Patients who died on the day of admission were excluded because they had less opportunity for transfer. Application of these exclusions left 184,295 patients from 4,765 hospitals.

Patients who underwent transfer were our primary study group of interest. We define "transfer" as occurring when a patient is admitted to one acute care hospital and discharged from a different hospital during an episode of care for an AMI. The CCP records for patients transferred into an index hospital contain the source of admission but no detailed information on pre-admission clinical course. For patients transferred from an index hospital, the records contain discharge destination but not detailed information on post-discharge course. However, vital status was available for all patients from Medicare administrative data, in particular at 30 days after hospitalization.

### Hospitals

Teaching hospitals were defined as those with an intern/bed ratio greater than zero[8], as assessed by merging the CCP and CMS administrative data sets. Hospital location was defined as rural or urban by metropolitan statistical area[23].

For each hospital, we derived a technology index (TI) as described below:

1. No Angiographic, PTCA or CABG capacity

2. Angiographic capacity only

3. Angiographic and PTCA capacity only

4. Angiographic, PTCA, and CABG capacity

### Process and outcome measures

We focused on four quality measures that were developed as part of the CCP: in-hospital administration of aspirin, beta-blockers, ACE- inhibitors and acute reperfusion to eligible candidates. We considered beta-blockers indicated for those patients who both lacked absolute contraindications and met inclusion criteria[20]. We considered aspirin and thrombolytic therapy indicated in patients who lacked both absolute and relative contraindications and met inclusion criteria. Definitions of contraindications and therapy groups are described further in Allison et al[24]. Because of significant interactions between receipt of acute reperfusion and aspirin, we created 5 mutually exclusive groups of patients: therapy category 0, those who received no therapy (reference group); therapy category 1, those who received no aspirin and no reperfusion but did receive angiotensin-converting enzyme (ACE) inhibitors and/or b-blockers; therapy category 2, those who received no reperfusion but did receive aspirin and/or ACE inhibitors and/or b-blockers; therapy category 3, those who received no aspirin but did receive reperfusion and/or ACE inhibitors and/or b-blockers; and therapy category 4, those who received aspirin and reperfusion and/or ACE inhibitors and/or b-blockers.

Patients transferred in from another acute care hospital were not considered eligible for reperfusion at the receiving hospital. Mortality at 30 days after admission was ascertained from HCFA administrative data. Patient co-morbidity and severity of illness were assessed by the Adapted Charlson Index[25] and by the APACHE II scale[26], respectively.

### Statistical analyses

Two separate analyses were conducted. First, we compared transferred and non-transferred patients. Because interhospital transfer is more common in rural hospitals we performed a second analysis comparing patients cared for in rural and urban hospitals. Differences between groups (transferred v non-transferred and rural v urban) were tested using the chi-square or Kruskal-Wallis test[27].

We first examined unadjusted 30-day mortality for each comparison group; transfer v non-transfer, and rural v urban. Predicted mortality was calculated using patient demographics, and other clinical predictors of mortality based on the work of Krumholz (age, gender, race, serum white blood count [WBC] on admission, serum creatinine, presence of heart failure on admission, cardiac arrest, and location of MI)[28].

Because transfer status and location of hospital was not randomly assigned in this patient population, there was substantial potential for confounding and selection bias. We chose to account for this by developing a propensity score for transfer and rural status. Propensity score analysis is a post-hoc statistical method that estimates treatment effect when subjects were not randomly assigned to treatment group. Propensity score analysis attempts to simultaneously control for all known patient factors that might be related to the outcome of interest. Joffe and Rosenbaum have described the rationale and methods underlying the use of propensity score analysis[29].

We constructed nonparsimonious logistic regression models in which inter-hospital transfer was a dependent variable and the variables in Table 1 were independent variables. These models made it possible to calculate a propensity score, indicating the likelihood that any individual patient would undergo interhospital transfer given all other known variables except 30-day mortality. The C-statistic of the logistic regression model used to generate the propensity score for transfer was 0.68 indicating a moderate ability to differentiate between transferred and non-transferred patients. The C-statistic represents the discriminative power of the logistic regression model. We performed a similar analysis for patients cared for in a rural hospital. The C-statistic of the logistic regression model used to generate the propensity score for rural hospital status was 0.57 indicating a fair ability to differentiate between rural and urban patients.

**Table 1:** Patient characteristics for transferred v non-transferred patients and rural v urban patients*

	Non-Transferred N = 132,765	Transferred N = 51,530		Urban N = 148,471	Rural N = 35,824	
Continuous Variables						

	Mean	Mean	p-value	Mean	Mean	p-value

Age	77.2	73.5	< 0.001	76.0	76.7	< 0.001
Charlson Score	0.72	0.55	< 0.001	0.67	0.68	0.06
Krumholz Predicted Mortality	0.19	0.15	< 0.001	0.18	0.18	0.07
APACHE Score	9.9	8.2	< 0.001	9.4	9.6	< 0.001
SBP on Admittance	144	141	< 0.001	142	146	< 0.001
Creatinine on Admission	1.4	1.3	< 0.001	1.4	1.4	0.12
WBC on Admission	11.0	10.6	< 0.001	10.9	10.8	< 0.001
						
Categorical Variables						

	%	%	p-value	%	%	p-value

Female	50	44	< 0.001	48	49	<0.001
African American	6	4	< 0.001	6	5	< 0.001
Terminal Illness	0.4	0.2	< 0.001	0.4	0.4	0.76
Diabetes	31	29	< 0.001	31	31	0.77
Chronic Renal Insufficiency	5	3	< 0.001	5	4	< 0.001
Hypertension	62	61	< 0.001	63	59	< 0.001
Malignancy	3	2	< 0.001	3	3	0.63
History of Heart Failure	24	13	< 0.001	21	22	< 0.001
History of CAD	40	36	< 0.001	39	37	< 0.001
History of Acute MI	32	28	< 0.001	31	31	0.08
History of PCTA	7	9	< 0.001	8	5	< 0.001
History of Bypass Surgery	13	12	< 0.001	13	11	< 0.001
Shock on Admittance	5	4	< 0.001	5	3	< 0.001
Cardiac Arrest	15	12	< 0.001	14	16	< 0.001
Heart Failure on Admission	51	37	< 0.001	48	46	< 0.001
Angina on Admission	2	3	< 0.001	2	3	< 0.001
Abnormal Rhythm on Admission	49	42	< 0.001	47	44	< 0.001
Anterior or Lateral MI	47	46	0.40	47	46	< 0.001

*Because the CCP includes such a large number of subjects, there are statistically significant differences that do not necessarily represent clinically significant differences.

We used the propensity score to randomly match transferred patients to non-transferred patients and rural patients to urban patients. Specifically, pairs of propensity scores were randomly matched using a greedy matching technique[30]. This technique randomly matched 44,175 transferred and non-transferred patients and 32,131 rural and urban patients.

For each comparison we then used a multivariable logistic regression model to adjust mortality analyses for patient demographics, severity of illness based on work by Krumholz[28], treatment according to a schema developed by Allison[24], hospital technology index, teaching status, and hospital size. We included in each analysis any additional covariant for which there had been a significant difference among our propensity-matched cohorts. All analyses were conducted using SAS version 9.00 (SAS Institute Inc., Cary, NC).

## Results

### Hospital and patient characteristics

Our study sample consisted of 184,295 patients from 4,765 hospitals. Overall, 51,530 AMI patients (28.0%) were transferred at some point during their hospital stay; 32,080 (17.4%) were transferred out of and 19,450 (10.6%) transferred into a CCP hospital. 35,824 (19.4%) patients were cared for in a rural hospital. Rural hospitals transferred 33.8% while urban hospitals only transferred 26.6%. Hospitals with lower technology index transferred a higher proportion of their patients (34% for low technology hospitals v 23% or high technology hospitals).

Baseline patient characteristics according to transfer status and hospital location are summarized in Table 1. Because the CCP is such a large database there are statistically significant differences that do not necessarily represent clinically significant differences. It is essential to consider both clinical and statistical significance when reviewing these tables. Table 2 describes treatment and crude mortality rates.

**Table 2:** Treatment and crude mortality among transferred v non-transferred patients and rural v urban patients

	Non-Transferred N = 132,765	Transferred N = 51,530		Urban N = 148,471	Rural N = 35,824	
Continuous Variables						

	Mean	Mean	p-value	Mean	Mean	p-value

Length of Stay	8.2	5.9	< 0.001	7.9	5.9	< 0.001
Categorical Variables						

	%	%		%	%	

ASA if Eligible	84	89	< 0.001	87	82	< 0.001
Beta Blocker if Eligible	50	57	< 0.001	54	44	< 0.001
ACE-I if Eligible	33	21	< 0.001	31	28	< 0.001
Thrombolytics if Eligible	53	71	< 0.001	58	62	< 0.001
Tobacco Cessation Counseling	6	6	0.02	6	5	< 0.001
**Died Within 30 Days**	**19**	**11**	**< 0.001**	**16**	**18**	**< 0.001**

### Transfer and mortality

Transferred patients were younger and had much lower predicted mortality than non-transferred patients. Transferred patients were more likely to be male, less likely to be African American, and less likely to have diabetes and a history of heart failure. Transferred patients were less likely to have heart failure or an abnormal heart rhythm on admission. Transferred patients had higher rates of use for of aspirin, beta-blockers, and thrombolytics (Table 2).

30,586 (16.6%) patients died within 30 days. Unadjusted 30-day mortality was lower among transferred patients (10.7% v 18.9%, p < .001). Based on systematically collected data for baseline demographics and medical risk factors a logistic regression model was used to generate a propensity score for transfer. The baseline demographic and medical risks comparing propensity matched transferred and non-transferred patients are shown in Table 3. As opposed to the entire population of CCP patients, these propensity-matched patients were well matched; the only clinically significant differences were that transferred patients had a slightly higher predicted mortality (15% v 14%, p = .007) and a slightly lower rate of diabetes (29.5% v 30.3%, p = .009). Transferred patients had a lower unadjusted 30-day mortality (10.7% v 12.5%. p < .001). This mortality advantage persisted after adjustment for patient demographics, therapy, and hospital characteristics. (O.R. for 30-day mortality = 0.80, 95% C.I. 0.76–0.84) (Table 4).

**Table 3:** Patient characteristics according to transfer status and hospital location in propensity matched groups

	Non-Transferred N = 44,175	Transferred N = 44,175		Urban N = 32,131	Rural N = 32,131	
Continuous Variables						

	Mean	Mean	p-value	Mean	Mean	p-value

Age	73.4	73.5	0.13	76.6	76.7	0.63
Charlson Score	0.56	0.55	0.17	0.68	0.68	0.59
Krumholz Predicted Mortality	0.14	0.15	<.01	0.18	0.18	0.60
APACHE Score	8.4	8.4	0.52	9.6	9.6	0.61
SBP on Admission	143	142	0.02	147	146	0.50
Creatinine on Admission	1.27	1.28	0.09	1.4	1.4	0.84
WBC on Admission	10.6	10.6	0.83	10.8	10.8	0.53
						
Categorical Variables						

	%	%	p-value	%	%	p-value

Female	43	43	0.39	49	49	0.89
African American	4	4	0.24	5	5	0.83
Terminal Illness	0.2	0.2	0.83	0.3	0.4	0.89
Diabetes	30	29	<.01	31	31	0.73
Chronic Renal Insufficiency	3	3	0.77	4	4	0.93
Hypertension	61	61	0.59	59	59	0.67
Malignancy	2	2	0.79	3	3	0.05
History of Heart Failure	13	13	0.08	23	23	0.36
History of CAD	37	36	0.07	38	37	0.39
History of Acute MI	28	28	0.95	31	31	0.28
History of PCTA	9	8	0.33	5	5	0.91
History of Bypass Surgery	13	12	<.01	11	11	0.38
Shock on Admission	4	4	0.14	3	3	1.00
Cardiac Arrest	13	12	0.01	15	15	0.10
Heart Failure on Admission	38	38	0.90	46	46	0.98
Angina on Admission	3	3	0.06	3	3	0.73
Abnormal Rhythm on Admission	42	41	0.18	44	44	0.67
Anterior or Lateral MI	47	47	0.85	46	46	0.59

**Table 4:** Odds ratios for 30-day mortality in propensity score matched patients across model groups

	Transferred v Non-Transferred (Referent)			Rural v Urban (Referent)		
	O.R.	95% C.I.		O.R.	95% C.I.	

Assigned Model Covariates						
Model Groups (vs Referrent)	**0.80**	**0.76**	**0.84**	**1.05**	**0.99**	**1.11**
Age	1.38	1.35	1.41	1.43	1.39	1.47
Ethnicity (African American as Referent)	0.89	0.79	1.01	0.87	0.77	0.98
Gender (Female as Referent)	1.14	1.09	1.20	1.12	1.07	1.18
Cardiac Arrest	6.66	6.33	7.02	6.23	5.91	6.58
Congestive Heart Failure	2.14	2.04	2.25	2.01	1.91	2.11
Systolic Blood Pressure on Admission	0.71	0.69	0.73	0.66	0.65	0.68
Serum Creatinine on Admission	1.20	1.18	1.22	1.23	1.20	1.26
White Blood Cell Count on Admission	1.26	1.24	1.29	1.25	1.22	1.28
Anterior or Lateral MI	1.49	1.42	1.56	1.51	1.44	1.58
Therapy (1 vs 0)	0.44	0.40	0.49	0.45	0.41	0.49
Therapy (2 vs 0)	0.29	0.27	0.32	0.30	0.28	0.33
Therapy (3 vs 0)	0.72	0.63	0.84	0.65	0.55	0.78
Therapy (4 vs 0)	0.24	0.22	0.26	0.25	0.23	0.28
Technology (1 vs 0)	0.98	0.92	1.05	1.06	0.99	1.13
Technology (2 vs 0)	0.99	0.85	1.15	1.17	0.99	1.37
Technology (3 vs 0)	0.91	0.84	0.98	1.02	0.94	1.11
Hospital Bed Size	0.99	0.97	1.03	0.99	0.95	1.02
Teaching Hospital (vs Non-Teaching)	0.89	0.83	0.94	0.93	0.87	0.99
C-Statistic	0.82			0.82		

O.R. for Age, SBP, Serum Creatinine, WBC, and Hospital Bed Size represent 1 standard deviation unit.

O.R. refers to Odds Ratio

C.I. refers to Confidence Interval

### Rural hospital and mortality

35,824 (19.4%) patients were cared for in a rural hospital. Rural patients were slightly older than urban patients, but had similar predicted mortality (Table 1). Rural patients were more likely to be female, less likely to be African American, had a higher rate of history of heart failure but had similar rates of diabetes. Rural patients were slightly less likely to have heart failure or an abnormal heart rhythm on admission. Rural patients had lower rates of use for aspirin, beta-blockers, and ACE-Inhibitors, but a higher rate of use for thrombolytic therapy (Table 2).

The baseline demographic and medical risks among propensity matched rural and urban patients are shown in Table 3. These propensity-matched patients were well matched. Rural patients had a higher unadjusted 30-day mortality (17.5% v 16.3%. p < .001). However, after adjustment for patient demographics, therapy, and hospital characteristics this mortality advantage disappeared. (O.R for 30-day mortality = 1.05, 95% C.I. 0.99–1.11) (Table 4).

## Discussion

We found that over one quarter of AMI patients were transferred during their hospital course. Transferred patients were significantly younger and more often white, male, with fewer co-morbid conditions and less severe disease. Transferred patients were also more likely to receive appropriate therapy. Transferred patients had lower unadjusted 30-day mortality than non-transferred patients. After a rigorous propensity analysis of nearly 90,000 propensity -matched patients, this mortality benefit persisted. Physicians decide to transfer patients for many explicit and implicit reasons. The transferring physicians in the CCP may have understood which patients would most benefit from transfer. Testing these hypotheses will require prospective data collected with more clinical detail than we have available in the current data.

We found that patients cared for in rural hospitals had slightly lower rates of treatment with 3 quality care measures (aspirin, beta-blockers, and ACE-Inhibitors), while they had a higher rate of treatment with thrombolytic therapy. This may be due to decreased availability of angioplasty in rural hospitals. After analysis of over 64,000 propensity-matched patients and adjustment for patient differences, treatment differences and hospital characteristics, patients cared for in a rural hospital had similar mortality as patients cared for in an urban hospital. The high rate of transfer among younger, healthier patients may partially account for the lower quality of care and worse outcomes ascribed to rural hospitals previously reported by others.

To our knowledge, this is the first national study of the characteristics and mortality of Medicare patients with AMI according to transfer status. Our results are consistent with those of Mehta and colleagues, who used CCP data only from the state of Michigan to examine the implications of patient transfer[15]. Mehta found that patients who were transferred from hospitals with lower technological capability to hospitals with higher technological capability tended to be younger, more likely to be white and male, and had lower predicted mortality.

The bulk of the literature on transfer of patients with AMI focuses exclusively on patients transferred for specific procedures [31-34]. For example, Straumann et al. evaluated prospectively the baseline characteristics and outcomes of AMI patients transferred to a tertiary referral center for primary PTCA and compared these patients with patients directly admitted to the same referral center[35]. They found that the patients who were transferred-in tended to be younger, more critically ill, more likely to be in cardiogenic shock or require resuscitation, but had similar mortality. Liem et al. compared transferred to non-transferred PTCA patients to evaluate treatment delay, infarct size and mortality[36]. They found that despite an average 43-minute treatment delay for transfers and larger infarct size, transferred and non-transferred patients had similar 6-month clinical outcomes. Andersen et al. recently reported that patients transferred for primary PTCA had better outcomes than non-transferred patients receiving thrombolytic therapy[37]. However, the benefit was solely in terms of decreased re-infarction and there was no statistically significant benefit to transfer in terms of mortality or stroke.

Our finding that there are major differences between transferred and non-transferred patients has particular relevance for the understanding of quality of care in rural hospitals. Previous studies have frequently deleted transferred patients from analysis. Because transferred patients tend to be younger, healthier, male, and have lower predicted mortality, comparisons between hospitals are subject to a significant bias against hospitals that transfer a higher proportion of AMI patients. Thiemann and Casale in separate reports found that rural and smaller hospitals had worse outcomes than urban and larger hospitals[16,17]. However, their studies deleted transferred patients from their analysis. After accounting for the numerous and large differences between transferred and non-transferred patients, we found that patients cared for in rural hospitals had similar outcomes to patients cared for in urban hospitals.

Although the reason for transfer is not documented in the CCP dataset, we can make some inferences based on the characteristics of hospitals transferring and accepting patients, and by the treatments administered to each group of patients. From our data it is clear that smaller, rural hospitals with less technological capacity are more likely to transfer patients to another institution. Larger, urban hospitals with the ability to perform cardiac catheterization, PTCA, and bypass surgery are less likely to transfer.

In a rural hospital without advanced cardiac services transfer may be viewed as a treatment option, just like the use of aspirin, beta-blockers, and thrombolytics. While there are evidence-based guidelines for the medical treatments of acute MI, there are no guidelines aiding the decision of whether to transfer a patient suffering an acute MI. Certainly, a patient who requires cardiac surgery or urgent angiography will benefit from transfer. Identification of other patient groups likely to benefit from transfer will provide guidance to the clinician faced with the decision whether or not to transfer a patient.

For health services research the issue surrounding the analysis of transferred patients is complex. There is disagreement about where to assign responsibility for outcomes. Because transfer is so common and may actually represent a treatment option, rather than an outcome, the "assignment of responsibility" may not be the most important question. The important question for hospitals without interventional cardiac services may be how to identify the patient who is most likely to benefit from transfer. Transfer rates have increased dramatically in the past decade making it even more important to understand the risks and benefits associated with transfer[4].

The major limitation of this study is that transfer and rural hospitalization were not randomly assigned. The use of observational studies to assess treatment effects and outcomes is controversial. Additionally, chart review has its own unique limitations[38]. Several recent publications point out that properly performed observational studies are unlikely to lead to misleading or inappropriate conclusions[38,39]. We performed propensity analysis that provided a robust adjustment for selection bias and confounding. However, propensity analysis can only adjust for measured variables. For example, the CCP does not collect data on socioeconomic factors that may be related to use of more invasive treatment[40] and may predispose patients to transfer as well as to improved survival. Due to missing data we were unable to match a small portion of the transferred and rural patients. However, our match rate of 86–90% is within the range of previously reported propensity analyses[41,42].

## Conclusion

We found that Medicare patients that underwent interhospital transfer during care of their acute MI had generally higher quality of care and lower mortality than non-transferred patients. However transferred patients were very different than non-transferred patients. These differences may partially account for the difference in mortality between rural and urban hospital previously reported. We found no difference in 30-day mortality between patients cared for in a rural or urban hospital. Deleting transferred patients from analysis may introduce significant bias. For patients living in a rural community it is reasonable for them to present to their local hospital, and the decision whether to transfer or not becomes a clinically important element in their care. Additional work is needed to better define characteristics of the patient and the health care system that might identify those most likely to benefit from transfer and methods to expedite transfer for those patients.

## Competing interests

The authors declare that they have no competing interests.

## Authors' contributions

JW conceived of the initial research question, participated in the design of the study, interpretation of the results and drafted the manuscript. NW, AG, RC, and OW provided statistical analysis and interpretation, reviewed results, and participated in manuscript preparation. CK and JA participated in the design of the study, coordinated analysis and manuscript preparation and provided leadership to the research team. All authors read and approved the final manuscript.

## Pre-publication history

The pre-publication history for this paper can be accessed here:



## References

[B1] Murray CJ, Lopez AD (1997). Mortality by cause for eight regions of the world: Global Burden of Disease Study. Lancet.

[B2] Centers for Disease Control and Prevention (CDC) (2001). Mortality from coronary heart disease and acute myocardial infarction – United States, 1998. MMWR – Morbidity & Mortality Weekly Report.

[B3] Wyatt S, Moy E, Levin R, Lawton K, Witter DJ, Valente EJ, Lala R, Griner P (1997). Patients transferred to academic medical centers and other hospitals: Characteristics, resource use, and outcomes. Acad Med.

[B4] Westfall JM, McGloin J (2001). Impact of double counting and transfer bias on estimated rates and outcomes of acute myocardial infarction. Med Care.

[B5] Gordon HS, Rosenthal GE (1996). Impact of interhospital transfers on outcomes in an academic medical center. Implications for profiling hospital quality. Med Care.

[B6] Bernard AM, Hayward RA, Rosevear J, Chun H, McMahon LF (1996). Comparing the hospitalizations of transfer and non-transfer patients in an academic medical center. Acad Med.

[B7] Romano PS, Zach A, Luft HS, Rainwater J, Remy LL, Campa D (1995). The California Hospital Outcomes Project: Using administrative data to compare hospital performance. Jt Comm J Qual Improv.

[B8] Ballard DJ, Bryant SC, O'Brien PC, Smith DW, Pine MB, Cortese DA (1994). Referral selection bias in the Medicare hospital mortality prediction model: Are centers of referral for Medicare beneficiaries necessarily centers of excellence?. Health Serv Res.

[B9] Clough JD, Kay R, Gombeski WR, Nickelson DE, Loop FD Mortality of patients transferred to a tertiary care hospital. Cleve Clin J Med.

[B10] Burney RE, Walsh DG (1988). Identification and transport of patients with acute myocardial infarction for thrombolytic therapy. Ann Emerg Med.

[B11] Rubenstein DG, Treister NW, Kapoor AS, Mahrer PR (1988). Transfer of acutely ill cardiac patients for definitive care. Demonstrated safety in 755 cases. JAMA.

[B12] Kaplan L, Walsh D, Burney RE (1987). Emergency aeromedical transport of patients with acute myocardial infarction. Ann Emerg Med.

[B13] Ryan TJ, Anderson JL, Antman EM, Braniff BA, Brooks NH, Califf RM, Hillis LD, Hiratzka LF, Rapaport E, Riegel BJ, Russell RO, Smith EE, Weaver WD (1996). ACC/AHA guidelines for the management of patients with acute myocardial infarction. A report of the American College of Cardiology/American Heart Association Task Force on Practice Guidelines (Committee on Management of Acute Myocardial Infarction). J Am Coll Cardiol.

[B14] Oude Ophuis TJ, Bar FW, Vermeer F, Krijne R, Jansen W, de Swart H, van Ommen V, de Zwaan C, Engelen D, Dassen WR, Wellens HJ (1999). Early referral for intentional rescue PTCA after initiation of thrombolytic therapy in patients admitted to a community hospital because of a large acute myocardial infarction. Am Heart J.

[B15] Mehta RH, Ruane TJ, McCargar PA, Eagle KA, Stalhandske EJ (2000). The treatment of elderly diabetic patients with acute myocardial infarction: Insight from Michigan's Cooperative Cardiovascular Project. Arch Intern Med.

[B16] Casale PN, Jones JL, Wolf FE, Pei Y, Eby LM (1998). Patients treated by cardiologists have a lower in-hospital mortality for acute myocardial infarction. J Am Coll Cardiol.

[B17] Thiemann DR, Coresh J, Oetgen WJ, Powe NR (1999). The association between hospital volume and survival after acute myocardial infarction in elderly patients. N Engl J Med.

[B18] Sheikh K, Bullock C (2001). Urban-rural differences in the quality of care for medicare patients with acute myocardial infarction. Arch Intern Med.

[B19] Bratzler DW, de Leon AC, Johnson MC, Oehlert WH, Slagle RC, Murray CK, Bumpus LJ, Webb The Cooperative Cardiovascular Project in Oklahoma. J Okla State Med Assoc.

[B20] Marciniak TA, Ellerbeck EF, Radford MJ, Kresowik TF, Gold JA, Krumholz HM, Kiefe CI, Allman RM, Vogel RA, Jencks SF (1998). Improving the quality of care for Medicare patients with acute myocardial infarction: Results from the Cooperative Cardiovascular Project. JAMA.

[B21] Ellerbeck EF, Jencks SF, Radford MJ, Kresowik TF, Craig AS, Gold JA, Krumholz HM, Vogel RA (1995). Quality of care for Medicare patients with acute myocardial infarction. A four-state pilot study from the Cooperative Cardiovascular Project. JAMA.

[B22] Public Health Service, U.S. Dept of Health and Human Services (1997). International Classification of Diseases, Ninth Revision, Clinical Modification.

[B23] U.S. Census Bureau (1994). Geographic Areas Reference Manual: U.S. Department of Commerce, Economics and Statistics Administration, Bureau of the Census.

[B24] Allison JJ, Kiefe CI, Weissman NW, Person SD, Rousculp M, Canto JG, Bae S, Williams OD, Farmer R, Centor RM (2000). Relationship of hospital teaching status with quality of care and mortality for Medicare patients with acute MI. JAMA.

[B25] Charlson ME, Pompei P, Ales KL, MacKenzie CR (1987). A new method of classifying prognostic comorbidity in longitudinal studies: Development and validation. J Chronic Dis.

[B26] Knaus WA, Draper EA, Wagner DP, Zimmerman JE (1985). APACHE II: A severity of disease classification system. Crit Care Med.

[B27] Rosner B (1995). Fundamentals of Biostatistics.

[B28] Krumholz HM, Chen J, Wang Y, Radford MJ, Chen YT, Marciniak TA (1999). Comparing AMI mortality among hospitals in patients 65 years of age and older: Evaluating methods of risk adjustment. Circulation.

[B29] Joffe MM, Rosenbaum PR (1999). Invited commentary: Propensity scores. Am J Epidemiol.

[B30] (1989). Massey JMTPVTW. 1985–94 National Center for health statistics.

[B31] Gore JM, Corrao JM, Goldberg RJ, Ball SP, Weiner BH, Aghababian RV, Dalen JE (1989). Feasibility and safety of emergency interhospital transport of patients during early hours of acute myocardial infarction. Arch Intern Med.

[B32] Bellinger RL, Califf RM, Mark DB, Weber RA, Collins P, Stone J, Phillips HR, German L, Stack RS (1988). Helicopter transport of patients during acute myocardial infarction. Am J Cardiol.

[B33] Zijlstra F, van't Hof AW, Liem AL, Hoorntje JC, Suryapranata H, de Boer MJ (1997). Transferring patients for primary angioplasty: A retrospective analysis of 104 selected high risk patients with acute myocardial infarction.

[B34] Connor SB, Lyons TJ (1995). A review of United States Air Force aeromedical evacuation of acute myocardial infarction patients in Europe. Mil Med.

[B35] Straumann E, Yoon S, Naegeli B, Frielingsdorf J, Gerber A, Schuiki E, Bertel O (1999). Hospital transfer for primary coronary angioplasty in high risk patients with acute myocardial infarction. Heart.

[B36] Liem AL, van't Hof AW, Hoorntje JC, de Boer MJ, Suryapranata H, Zijlstra F (1998). Influence of treatment delay on infarct size and clinical outcome in patients with acute myocardial infarction treated with primary angioplasty. J Am Coll Cardiol.

[B37] Andersen HR, Nielsen TT, Rasmussen K, Thuesen L, Kelbaek H, Thayssen P, Abildgaard U, Pedersen F, Madsen JK, Grande P, Villadsen AB, Krusell LR, Haghfelt T, Lomholt P, Husted SE, Vigholt E, Kjaergard HK, Mortensen LS (2003). A comparison of coronary angioplasty with fibrinolytic therapy in acute myocardial infarction. N Engl J Med.

[B38] Concato J, Shah N, Horwitz RI (2000). Randomized, controlled trials, observational studies, and the hierarchy of research designs. N Engl J Med.

[B39] Benson K, Hartz AJ (2000). A comparison of observational studies and randomized, controlled trials. N Engl J Med.

[B40] Carlisle DM, Leake BD (1998). Differences in the effect of patients' socioeconomic status on the use of invasive cardiovascular procedures across health insurance categories. Am J Public Health.

[B41] Seeger JD, Walker AM, Williams PL, Saperia GM, Sacks FM (2003). A propensity score-matched cohort study of the effect of statins, mainly fluvastatin, on the occurrence of acute myocardial infarction. Am J Cardiol.

[B42] Gum PA, Thamilarasan M, Watanabe J, Blackstone EH, Lauer MS (2001). Aspirin use and all-cause mortality among patients being evaluated for known or suspected coronary artery disease: A propensity analysis. JAMA.

